# One-Year Remission Rate of Chronic Headache Comparing Video and Face-to-Face Consultations by Neurologist: Randomized Controlled Trial

**DOI:** 10.2196/30151

**Published:** 2021-12-13

**Authors:** Svein Ivar Bekkelund, Kai Ivar Müller

**Affiliations:** 1 Department of Clinical Medicine The Arctic University of Norway Tromsø Norway

**Keywords:** chronic headache, remission, video consultation, telemedicine, eHealth, digital consultation, consultation, treatment, follow-up, RCT, randomized controlled trial

## Abstract

**Background:**

Chronic headache causing severe headache-related disability for those affected by the disease is under- or misdiagnosed in many cases and therefore requires easy access to a specialist for optimal health care management.

**Objective:**

The goal of the research is to determine whether video consultations are noninferior to face-to-face consultations in treating chronic headache patients referred to a specialist in Northern Norway.

**Methods:**

Patients included in the study were recruited from general practice referrals to a specialist at a neurological department in Northern Norway (Tromsø) and diagnosed according to the International Headache Society classification system. In a randomized controlled design, the 1-year remission rate of chronic headache (change from ≥15 to <15 headache days per month during the last 3 months), patient satisfaction with a specialist consultation, and need for follow-up consultations by general practitioners were compared between groups consulted by video and face-to-face in a post hoc analysis. Data were collected by interview (baseline) and questionnaire (follow-up).

**Results:**

From a baseline cohort of 402 headache patients consecutively referred from general practice to a specialist over 2.5 years, 58.0% (233/402) were classified as chronic headache and included in this study. Response rates were 71.7% (86/120) in the video group and 67.3% (76/113) in the face-to-face group. One-year remission from chronic headache was achieved in 43.0% (37/86) in the video group and 39.5% (30/76) in the face-to-face group (*P*=.38). Patient satisfaction with consultations were 86.5% (32/37; video) and 93.3% (28/30; face-to-face; *P*=.25). A total of 30% (11/37) in the video group and 53% (16/30) in the face-to-face group consulted general practitioners during the follow-up period (*P*=.03), and median number of consultations was 1 (IQR 0-13) and 1.5 (IQR 0-15), respectively (*P*=.19).

**Conclusions:**

One-year remission rate from chronic headache was about 40% regardless of consultation form. Likewise, patient satisfaction with consultation and need for follow-up visits in general practice post consultation was similar. Treating chronic headache patients by using video consultations is not inferior to face-to-face consultations and may be used in clinical neurological practice.

**Trial Registration:**

ClinicalTrials.gov NCT02270177; https://clinicaltrials.gov/ct2/show/NCT02270177

## Introduction

### Background

Chronic headache is a condition that transforms from primary headaches and is mainly identified as chronic migraine and chronic tension-type headache affecting about 1% to 2% and 2% of the population, respectively [1-3]. Lack of diagnostic biomarkers is a major challenge, and the chronic headache diagnosis (presence of headache 15 or more days per month for the last 3 months) is made by using a structured interview that relies on a validated diagnostic classification system [4]. Headache burden in the population is high and has not improved at the population level over time [5,6]. Preferably, headache should be correctly classified and treated at earliest to minimize risk of becoming chronic and development of associated conditions such as psychiatric symptoms [7], comorbid pain [8], increased costs for patients and the society [9], and impaired work performance [10], which are some known consequences that may burden chronic headache patients further. Depression, anxiety, sleep problems, stress, medication overuse, and low degree of headache self-management were associated with poorer prognosis of chronic headache in randomized controlled trials (RCTs) and prospective cohorts as summarized in an American review [11]. It has long been known that headache syndromes are underdiagnosed and undertreated, especially migraine [12,13]. There are many possible reasons for that. Professional headache care needs to be better coordinated with general health practice [14,15] and is further challenged by variable access to headache specialists [16]. In a group of 1254 chronic migraine sufferers, 40% had consulted a headache specialist, but only 4.5% reported that they additionally received correct diagnosis and treatment [13]. Knowledge about how alternative specialist consultations using information and communication technology may be used to treat patients with difficult headache is limited. In less populated areas where a secondary health service such as a general neurological department is the only alternative to primary health care, telemedicine might be effective. The main hypothesis of this study was that treating chronic headache patients referred to a neurological department by using video consultations is not inferior to traditional face-to-face consultations.

## Methods

### Study Design and Hypotheses

The results from this study are based on post hoc analyses from a previous open-label randomized clinical trial where a larger group (n=409) of heterogeneous headache patients referred from general practitioners (GPs) to specialists were assigned to either video or face-to-face (in-office) consultations to study cost, feasibility, and clinical aspects [17]. The trial was registered at ClinicalTrials.gov [NCT02270177]. The group of patients with chronic headache from that cohort were selected for this study to test the following primary hypothesis: providing treatment to chronic headache patients by specialist video consultations is noninferior to face-to-face specialist consultations with respect to 1-year remission rate of chronic headache (change from ≥15 to <15 headache days per month during the last 3 months). Secondary hypotheses were (1) patient satisfaction with specialist consultation, (2) frequency of chronic headache patients visiting GP for headache in the 12 months postconsultation, and (3) median number of headache-related consultations at GPs in the 12 months posttreatment, all end points postulated to be indifferent between the groups. The CONSORT-eHEALTH (Consolidated Standards of Reporting Trials of Electronic and Mobile Health Applications and Online TeleHealth) was used as a guide in describing the scientific study method [18].

### Study Population and Randomization

**Figure 1:**
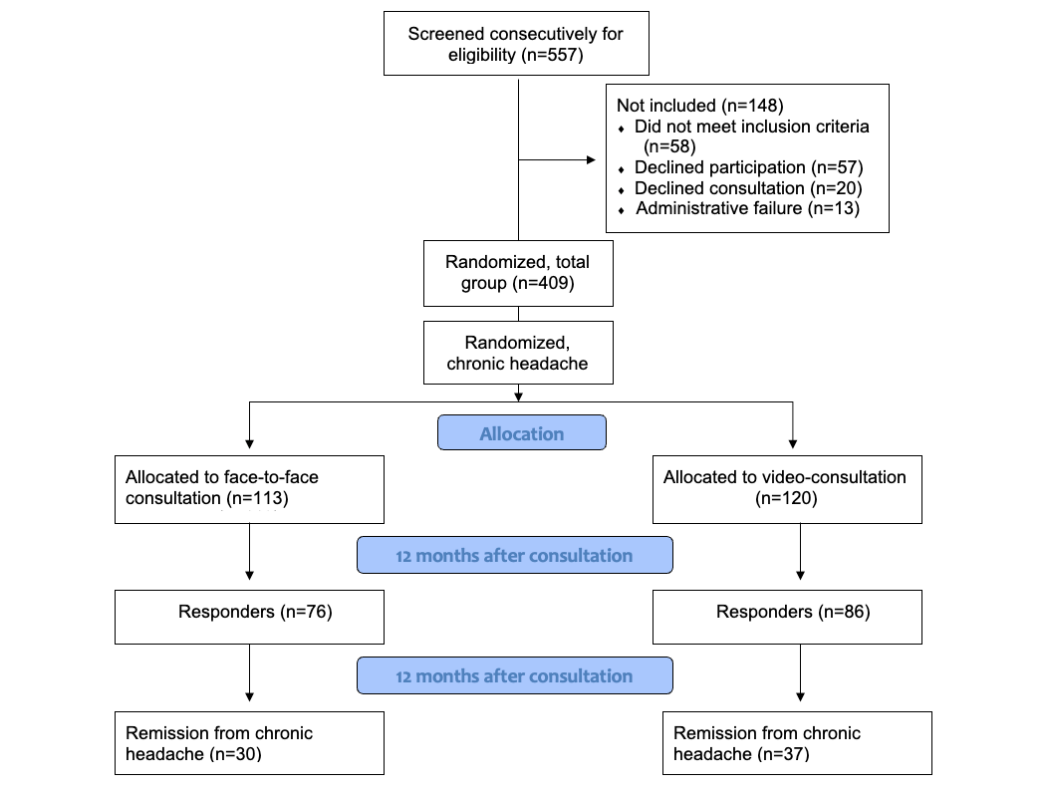
Flowchart of patients with chronic headache referred to neurologists from general practitioners for headache fulfilling the study inclusion criteria.

Patients were consecutively identified, screened, randomized, and consulted for 2.5 years (September 30, 2012, to March 30, 2015). Of the included patients, 58.0% (233/402) of patients were classified to have chronic headache and included in the study (Figure 1).

Selection criteria were as follows: (1) patients referred from GP to neurologist for headache, (2) fulfilling the classification criteria for chronic headache without evidence of secondary headache (ie, headaches classified as primary headache without specific causes [4] except patients with suspected medication overuse headache), (3) Norwegian speaking men and women aged 16 to 65 years, (4) not having visited a neurologist for headache within 2 years prior to consultation, and (5) waiting time from referral to consultation 4 months or less.

A nurse welcomed the participants at the entrance of the neurological department at the Tromsø University Hospital, checked the patient’s self-administered prefilled forms and participation consent and called the randomization administrator at the hospital (Centre for Quality Improvement and Development). Participants were block-randomized by using an Rnd function in Access (Microsoft Corp), and thereafter guided to an examination room for face-to-face consultation (traditional group) or to the video conference room located next to the department (video group). Video consultations were performed by using a video conference system including a C40 Integrator package (Cisco Systems Inc) with dual display option and Touch Control Device for C Series, C40 Integrator Multisite (Cisco Systems Inc), Precision HD 1080*P* 12× camera (Cisco Systems Inc), X551S 55” LED (Sharp NEC Display Solutions of America Inc) monitor, ceiling microphones (Audio-Technica Inc), and JBL LSR2325P (Harman International Industries) active speakers installed in the video conference room providing 2-way video and audio communication between patient and specialist. The neurologists consulted the patients from 2 other offices via a EX60 unit (Cisco Systems Inc) with an in-touch panel. Traditional face-to-face consultations were performed in the same offices. The study nurse confirmed that the visual and audio devices worked and informed the patients about the location of the web camera and microphone and where to sit. The nurse also provided a short training and assured optimal communication with the specialist. Two experienced neurologists (KIM and SIB) performed clinical consultations without neurological examinations but with additional checklists for inclusion criteria, diagnostic classification, and a standardized interview which were developed before the trial without further development during the trial. Further details are published elsewhere [19].

### Data Collection and End Points

Data were obtained by structured interview at baseline and via questionnaire at 1-year follow-up. The prefilled forms included a Headache Impact Test–6 (HIT-6) measuring 6 items of headache impact (pain, social, role and cognitive functioning, vitality, and psychological distress). Every question was answered by never, rarely, sometimes, very often, or always, and each answer scored 6, 8, 10, 11, or 13 points, respectively [20]. Pain intensity using a horizontal visual analog scale (VAS) ranging from 0 to 10 (0 = no pain, 10 = worst possible pain) was used in conjunction with HIT-6 [21]. Clinical and headache characteristics including comorbidity and diagnosis according to International Classification of Headache Disorders–2 [4] were recorded. Also, inaccurate headache diagnosis (ie, diagnostic disagreement between specialist and diagnoses reported in the electronic referral letter) were registered. The follow-up questionnaire recording demographics, clinical and headache characteristics, and end point variables was sent to the patients (with a reminder 2 weeks later to the nonresponders) either through an online survey service (Questback) or by postal letter. To classify chronic headache, the patients responded to number of headache days per month for the last 3 months.

Secondary end points were recorded from the patients’ registration form as follows: “Where you satisfied with the consultation?” (yes or no), “Have you consulted your GP for headache after the specialist consultation?” (yes or no), and “Number of headache consultations with GP after the specialist consultation.” Also, use of painkillers, triptans, and preventive headache drugs used in the last month were recorded.

### Ethics

Oral and written consent were obtained from all participants before study entrance. The Norwegian National Committee for Medical and Health Research Ethics approved the study (number 2009/1430/REK).

### Statistical Analyses

Data were analyzed with SPSS (version 27, IBM Corp). Descriptive variables are compared between the randomized groups and presented as mean and standard deviation or median and interquartile range in skewed distributed data (number of GP consultations). Consequently, comparisons between groups were analyzed by independent Student *t* test or Mann-Whitney *U* test, respectively; all 2-sided with *P*<.05 selected as level of statistical significance. Categorical variables were presented as numbers and percentages while groups were compared by using chi-square tests.

## Results

Patients’ characteristics were similar for both video and traditional consultations in all aspects including education and headache characteristics except for younger age in the video group (Table 1). A majority (174/233, 74.7%) had migraine as primary headache, and about one-third (79/233, 33.9%) used analgesic drugs and/or triptans or other headache-specific medication (Table 1). Consultation duration was shorter in the video group (Table 1). Inaccurate headache diagnosis (diagnostic disagreement between specialist and diagnoses reported in the electronic referral letter) are presented in Table 2. Comparisons between the video group and the face-to-face group were insignificant with respect to renewed headache diagnosis and preventive treatment given by the specialist (Table 2). The specialist prescribed preventive medication to 50% to 70% of the patients, and the group with chronic headache remission was treated similarly regardless of consultation form in that respect (Table 2). The main outcome was 43.0% (37/86) 1-year chronic headache remission rate in the video group compared to 39.5% (30/76) in the traditional group (*P*=.38; Table 3). Number and frequency of patients satisfied with consultations were 86.5% (32/37; video) and 93.3% (28/30; face-to-face; *P*=.25; Table 3). GP consultations (numbers and frequencies of patients and median numbers of consultations) are presented in Table 3. More patients treated traditionally (16/30, 53.3%) reported that they had consulted a GP for headache in the follow-up period (Table 3). No end points were otherwise statistically significantly different between the 2 groups. Neither were there any differences in changes in the HIT-6 and VAS scores from baseline to 1-year assessment between the 2 groups (Table 2).

**Table 1:** Clinical characteristics in randomized groups of patients referred to specialist for chronic headache consulted by video or traditionally.

Characteristic			Chronic headache at baseline							Remission from chronic headache at 12 months				
			Video (n=120)		Face-to-face (n=113)		*P* value		Video (n=37)			Face-to-face (n=30)		*P* value
One-year response, n (%)			86 (71.7)		76 (67.3)		.52		—^a^			—		—
Females, n (%)			86 (71.7)		84 (74.3)		.66		30 (72.1)			21 (70.0)		.39
Age (years), mean (SD)			35.2 (12.8)		40.0 (13.7)		.006		38.3(12.4)			41.2 (14.6)		.38
Education (years), mean (SD)			13.2 (2.9)		14.0 (3.1)		.07		13.3 (2.7)			13.9 (3.0)		.42
Sick leave (headache, weeks), n (%)			42 (35.0)		43 (38.1)		.68		11 (29.7)			12 (40.0)		.58
Waiting time to specialist (days), mean (SD)			59.0 (29.0)		55.2 (26.1)		.29		58.7 (25.5)			46.9 (23.5)		.06
Consultation duration (minutes), mean (SD)			40.2 (9.8)		46.5 (13.0)		<.001		41.0 (8.1)			45.8 (8.8)		.02
BMI (mg/m^2^), mean (SD)			27.1 (5.5)		26.9 (5.7)		.79		27.8 (4.5)			28.6 (7.5)		.35
Obesity, BMI ≥30, n (%)			31 (25.8)		29 (25.7)		>.99		27 (73.0)			20 (66.7)		.58
Without comorbidity, n (%)			62 (51.7)		52 (46.0)		.54		18 (48.6)			13 (43.3)		.81
Chronic neck pain, n (%)			56 (46.7)		57 (50.4)		.60		20 (54.1)			14 (46.7)		.63
Insomnia, n (%)			80 (66.7)		72 (63.7)		.68		9 (24.3)			10 (33.3)		.43
Hypertension, n (%)			11 (9.2)		17 (15.0)		.23		5 (13.5)			4 (13.3)		>.99
Age at headache onset (years), mean (SD)			24.4 (14.3)		27.7 (14.7)		.09		26.1 (15.3)			30.2 (15.8)		.29
Headache duration (years), mean (SD)			12.2 (12.8)		13.6 (14.6)		.35		13.2 (13.2)			15.3 (16.0)		.58
**Chronic headache subtype^b^, n (%)**														
	Migraine	90 (75.0)		84 (74.3)		>.99			31 (83.8)		23 (76.6)		.79	
	Tension-type	23 (19.2)		28 (24.8)		.34			6 (16.2)		6 (20.0)		—	
	Other	7 (5.8)		1 (0.9)		—			0 (0)		1 (3.3)		—	
Medication ≥15 days/month^c^, n (%)			39 (32.5)		40 (35.4)		.68		8 (21.6)			3 (10.0)		—

^a^Not applicable.

^b^Most prominent headache subtype given by specialist.

^c^Use of painkillers and/or triptans ≥15 days per month last 3 month.

**Table 2:** Diagnostic changes and preventive chronic headache treatment given by neurologist. Comparisons between groups of patients randomized to either video or traditional consultations.

Variable		Persistent chronic headache at 12 months			Remission from chronic headache at 12 months		
		Video (n=49)	Face-to-face (n=46)	*P* value	Video (n=37)	Face-to-face (n=30)	*P* value
New headache diagnosis, n (%)		15 (30.6)	9 (19.6)	.25	9 (24.3)	9 (30.0)	.78
**Preventive treatment, n (%)**		26 (53.1)	29 (63.0)	.41	26 (70.3)	21 (70.0)	>.99
	Antihypertensive	9 (18.4)	5 (10.9)	—^a^	9 (24.3)	3 (10.0)	—
	Antiepileptic	6 (12.2)	7 (15.1)	—	7 (18.8)	6 (20.0)	—
	Antidepressant	11 (22.5)	17 (37.0)	—	10 (27.0)	12 (40.0)	—
Triptans, n (%)		19 (38.8)	14 (30.4)	.55	11 (29.7)	6 (20.0)	.41

^a^Not applicable.

**Table 3:** Remission rates from chronic headache (primary end point), patient’s satisfaction with consultation and general practitioner consultations (secondary end points), headache-related symptoms, and therapy in the 12 months after specialist consultation. Patients randomized to either video or traditional consultations.

Variables	Persistent chronic headache at 12 months				Remission from chronic headache at 12 months		
	Video (n=49)	Face-to-face (n=46)	*P* value	Video (n=37)		Face-to-face (n=30)	*P* value
Remission rate from CH^a^ (%)^b^	—^c^	—	—	37/86 (43.0)		30/76 (39.5)	.38
Persistent CH (%)^b^	49/86 (57.0)	46/76 (60.5)	.38	—		—	—
Patient satisfaction with consultation, n (%)	42 (85.7)	41 (89.1)	.41	32 (86.5)		28 (93.3)	.25
GP^d^ consultations, n (%)	29 (59.2)	20 (43.5)	.12	11 (29.7)		16 (53.3)	.03
GP consultations, median (IQR range)	2 (0-11)	1 (0-11)	.04	1 (0-13)		1.5 (0-15)	.19
HIT^e^ -6, baseline, mean (SD)	64.0 (5.6)	64.9 (4.6)	.38	66.0 (3.9)		63.7 (6.4)	.09
HIT-6 after 1 year, mean (SD)	58.3 (8.8)	61.6 (7.8)	.10	59.9 (10.5)		59.2 (8.2)	.98
∆HIT-6, mean (SD)	5.7 (9.3)	3.3 (8.7)	.08	5.5 (12.4)		5.8 (9.6)	.92
VAS^f^, baseline, mean (SD)	6.9 (2.3)	6.9 (2.1)	.95	7.0 (2.1)		6.8 (2.0)	.66
VAS after 1 year, mean (SD)	5.2 (2.8)	6.6 (2.0)	.02	5.3 (2.8)		5.2 (3.2)	.94
∆VAS, mean (SD)	1.7 (3.8)	0.3 (3.5)	.01	1.7 (3.3)		1.6 (3.5)	.80
Analgesic use, n (%)	38 (77.6)	39 (84.8)	.44	34 (91.9)		29 (96.7)	.62
Medication ≥15 days/month^g^, n (%)	27 (55.1)	23 (50.0)	.48	8 (21.6)		3 (10.0)	—

^a^CH: chronic headache.

^b^Calculated by using response rates (per protocol analyses) as reference.

^c^Not applicable.

^d^GP: general practitioner.

^e^HIT: headache impact test.

^f^VAS: visual analog scale.

^g^Use of painkillers and/or triptans ≥15 days per month last 3 months.

When taken data from the groups together (pooled data), the comparisons between baseline and status after 12 months were as follows: remission rate from chronic headache was 41.4% (67/162) and numbers visiting GPs were 30.2% (49/162) of those with persisting chronic headache and 40.3% (27/67) in the chronic headache remission group (*P*=.41). Median numbers of GP consultations were 1.0 (IQR 0-15) and 2.0 (IQR 0-11), respectively (*P*=.25). The rate of participants using analgesic medication or triptans ≥15 days per month declined from 52.6% (50/95) to 16.4% (11/67) 1-year post consultation.

## Discussion

### Principal Findings

By managing new referred chronic headache patients at a secondary neurological center, the 1-year results from this post hoc RCT showed that consulting a neurological specialist by using video were equivalent to face-to-face consultations. Thus, we found no significant differences in remission rate from chronic headache, patient satisfaction with consultation, or GP visits due to headache conducted in the 1-year follow-up period. This study provides evidence to support specialist video consultations as a good alternative to face-to-face consultations in treating patients with chronic headache.

### Comparison With Prior Work

There are no previous studies comparing consultation forms in treating chronic headache by a specialist, but in an earlier RCT the group of chronic headache patients randomized to an internet-delivered self-managing relaxation program (n=39) improved by 47% on measures of self-reported headache symptoms compared to an equivalent control group recruited from the waiting list with symptom monitoring only [22]. That study documents the usefulness of communicating via electronic devices as an alternative to face-to-face consultations in treating difficult headache such as chronic headache in line with this study. Likewise, the 1-year treatment response is comparable between the studies (47% vs 43%) despite different treatment methods and outcomes [22]. A smaller RCT by Friedman et al [23] randomized 18 patients with severe migraine to video consultations and 12 to in-office visits in a tertiary headache center. Improvement in headache burden and number of headache days were not different between the groups, and the authors concluded that video consultations were as effective as in-office visits. Furthermore, the consultation time was shorter in the telemedicine cohort as in this study (Table 1) indicating that telemedicine is effective for physicians in treating difficult headache [23].

In our study, approximately 40% of the chronic headache patients had remitted 1-year postconsultation while about 60% persisted with chronic headache. This rate of remission is somewhat lower than a previous longitudinal study that showed a 40% persistent rate at 1 year and 25% at 2-year follow-up [24]. Medication overuse was associated with chronicity in that study, which is also indicated here, as the rate of participants using analgesic medication or triptans ≥15 days per month declined from 52.6% to 16.4%. Similar findings are also demonstrated in population-based studies [25]. RCTs as a method to investigate different neurological outpatient management are in general few [26,27], but use of telemedicine was equivalent to face-to-face consultations with specialist as far as number of consultations [28]. Teleneurology is nevertheless widely used in clinical practice [29-31] with favorable results from the patient perspective (time- and money-saving, communication, perceiving good care, and future preference) [31,32]. From a specialist point of view (n=135 specialists), headache and follow-up consultations were well suited for telemedicine [33].

In general, RCTs in eHealth are few despite occurrence of the COVID-19 pandemic situation, which has demonstrated a need for more evidence-based knowledge about the use of digital health technology in evaluating treatment effect, safety, and other aspects of patient management [34-37]. Patient education programs [38,39]; evaluation of psychological distress using teletechnology in diabetes [40]; use of mobile in suboptimal health [41], surgery care, and follow-up [42-44]; aphasia [44]; HIV consultations [38]; cancer symptom monitoring [45]; motor and cognitive function in stroke [46]; and COVID-19 follow-up [47] are areas where RCTs are used. Moreover, this study agrees with previous telemedicine RCTs in the same area reporting positive outcomes in treating diabetic foot ulcer [48] and in a follow-up study of orthopedic patients [49]. Thus, the RCT design is the main advantage of this study, especially since it is the first one to compare consultation forms with specialist in chronic headache where one treatment arm is based on teletechnology.

### Limitations

This post hoc study containing a 53% sample of the original cohort of headache sufferers may be prone to statistical type 2 failure due to risk of underpowered sample size, although the video and traditional consultation groups were similar with respect to group sizes and most of the social and clinical characteristics reflecting a design resistant to selection bias. Moreover, such a study lacks a placebo group and blinding, which would have optimized the evidence further. Awareness of the fact that this study compares different consultation forms and not specific treatment options should be emphasized. Interim analyses comparing additional clinical information between patient groups within the 1-year follow-up period might extend the knowledge about patient experiences with video consultations and should be performed in future studies. Additionally, consecutively including patients from clinical practice and a relatively low dropout rate accounts for acceptable generalizability.

### Conclusions

This RCT of video consultations for new referrals of chronic headache patients demonstrated that chronic headache remission rate, patient satisfaction with specialist consultation, and GP consultations for headache performed during follow-up were equivalent between the video group and the face-to-face group. This study adds to the documentation of eHealth in consulting headache patients by specialist.

## Appendix

Multimedia Appendix 1 CONSORT-eHEALTH checklist (V 1.6.1).

## Abbreviations

CONSORT-EHEALTH: Consolidated Standards of Reporting Trials of Electronic and Mobile Health Applications and Online TeleHealth
GP: general practitioner
HIT-6: Headache Impact Test–6
RCT: randomized controlled trial
VAS: visual analog scale
